# Proliferative Effect of Proanthocyanidins on HGF-1 and HPDLF Cells: An In Vitro Study

**DOI:** 10.3390/medicina61122098

**Published:** 2025-11-25

**Authors:** Evelina Alkimavičienė, Nomeda Basevičienė, Arvydas Strazdauskas, Rasa Banienė, Nijolė Savickienė

**Affiliations:** 1 Department of Dental and Oral Pathology, Lithuanian University of Health Sciences, 44307 Kaunas, Lithuania; nomeda.baseviciene@lsmu.lt; 2 Department of Biochemistry, Medical Academy, Lithuanian University of Health Sciences, 44307 Kaunas, Lithuania; arvydas.strazdauskas@lsmu.lt (A.S.); rasa.baniene@lsmuni.lt (R.B.); 3 Department of Pharmacognosy, Medical Academy, Lithuanian University of Health Sciences, 44307 Kaunas, Lithuania; nijole.savickiene@lsmu.lt

**Keywords:** periodontitis, fibroblasts, proanthocyanidins, chlorhexidine, in vitro techniques

## Abstract

*Background and Objectives:* The use of proanthocyanidins (PACNs) alongside standard periodontal treatment procedures can improve periodontal and peri-implant tissue healing. The present study aimed to evaluate the effect of different concentrations of *Pelargonium sidoides* root extract (PSRE) on periodontal tissue proliferation in comparison with chlorhexidine digluconate (CHX). *Materials and Methods*: A cell culture study was performed using human gingival fibroblast (HGF-1) and human periodontal ligament fibroblast (HPDLF) lines. The HGF-1 cell line was exposed to CHX (the gold standard treatment in periodontal diseases) and PSRE at concentrations of up to 800 μg/mL, which were compared with negative controls. HGF-1 viability and proliferation were evaluated using fluorescence tests and the PrestoBlue assay, respectively. In addition, the cell proliferation induction ability of PSRE was evaluated by treating HGF-1 and HPDLF cells with PSRE at 25 and 50 μg/mL concentrations and measuring the TGFβ-1 levels using TGFβ-1 ELISA. *Results*: When comparing the effects of the 25 μg/mL PSRE treatment to the control, a statistically significant difference in HGF-1 cell growth was observed (0.297 ± 0.048 (mean ± SE) and 0.203 ± 0.01, respectively; *p* = 0.006). The strongest cytotoxic effect on HGF-1 cells was observed with CHX (0.007 ± 0.006, *p* < 0.001 vs. control). The HGF-1 and HPDLF cells showed statistically significant increases in TGFβ-1 levels when treated with PSRE at 25 and 50 μg/mL compared with the control (352.38 ± 31.32 (mean ± SE) and 330.99 ± 26.53 versus 161.07 ± 15.11 in HGF-1 cells; 397.53 ± 18.1 and 399.91 ± 27.61 versus 137.7 ± 16.54 in HPDLF cells, *p* < 0.001). Additionally, no negative effects were detected at low PSRE concentrations (less than 100 μg/mL). *Conclusions*: The results of this study suggested that PACNs may promote HGF-1 and HPDLF cell proliferation. In contrast, CHX showed cytotoxic effects.

## 1. Introduction

Proanthocyanidins (PACNs), a group of plant-based flavonoids, are well-known for a wide variety of beneficial biological properties, including maintaining periodontal and peri-implant health [1,2]. PACNs are found in different plant parts, including the fruits, leaves, roots, and seeds. The chemical structure of PACNs depends on the plant species [3]. This study focused on *Pelargonium sidoides* root extract (PSRE), in which, PACNs are the main components and are responsible for the extract’s effects [4].

Investigation of PSRE is an ongoing project of our research team, and we have published several studies on PSRE [5,6,7] showing that PSRE has a unique antibacterial property that selectively targets periodontal and peri-implant pathogenic strains, such as *Porphyromonas gingivalis*, while preserving the beneficial oral commensal strain *Streptococcus salivarius* [5]. This specific feature may be useful in the treatment of periodontal and peri-implant diseases. Therefore, the current study focused on PSRE as a source of PACNs.

The specific phytochemical profile of PACNs influences their biological effects. Since PACNs are oligomeric flavonoids, formed during the polymerization of flavan-3-ol monomers (catechin, epicatechin, etc.), the exact structure of the PACN depends on the type of flavan-3-ol, the degree of polymerization, the spatial configuration, etc. [3]. The type and location of structural units may affect their antioxidative capacities, stability, and bioavailability: different compositions may have differing efficacies in scavenging free radicals [8]. The degree of polymerization can have an impact on biological activity: short-chain PACNs are rapidly absorbed in the gastrointestinal tract while long-chain PACNs show strong anti-inflammatory effects [9,10]. Moreover, the positioning of hydroxyl groups in PACNs can influence their ability to neutralize reactive oxygen species, affecting their overall antioxidant capacity [11]. In addition, the hydroxyl groups form hydrogen bonds with other molecules, thereby modulating the activity of NF-κB and other signaling pathways that are responsible for the synthesis of proinflammatory mediators [12]. PACNs have different structural modifications (e.g., esterification) that can change their biological properties and activities [13]. Differences in their structures have significant effects on their ability to bind to specific receptors, which in turn impacts their activity on cells and tissues and general effectiveness. Overall, the specific phytochemical profile of PACNs directly impacts their biological activities (e.g., antioxidative and anti-inflammatory effects). Therefore, understanding these details is crucial for their therapeutic application.

The anti-inflammatory [14,15], antibacterial [16], and antioxidative properties [17,18,19] of PACNs are well established. Studies have shown that PACNs can decrease alveolar bone loss [2,20,21], inhibit collagen destruction [22,23], increase bone regeneration [24,25,26], and promote the osseointegration of dental implants [27]. These capacities are crucial to enhancing the healing process and stimulating periodontal tissue formation to promote periodontal and peri-implant tissue regeneration.

The current study focused on the ability of PSRE to induce the proliferation of HGF-1 and HPDLF cells through analyzing TGFβ-1 (transforming growth factor beta 1) levels using ELISA. TGFβ-1 is a growth factor that is involved in periodontal ligament cell regeneration [28,29,30] and plays a significant role in regulating fibroblast proliferation and differentiation and promoting extracellular matrix formation and collagen synthesis, which are critical for periodontal health. This growth factor is released into healing wounds (especially in the oral cavity) to stimulate several types of cells (fibroblast and osteoblasts) to proliferate [31].

To the authors’ knowledge, no in vitro study has been performed to evaluate the proliferative effect of PACNs from PSRE on human oral fibroblasts (human gingival and periodontal ligament fibroblasts). Thus, this study explored the ability of PSRE to induce proliferation of these cells.

## 2. Materials and Methods

### 2.1. Proanthocyanidin-Rich Material

Commercially available *PSRE* (Frutarom Switzerland Ltd., Rutiwisstrasse 7 CH-8820 Wadenswil, batch No. 0410100, Reinach, Switzerland) was diluted with a 30% polyethylene glycol solution. PSRE stock solutions were freshly prepared for each assay.

### 2.2. Cell Culture and Growth Conditions

Commercially available HGF-1 and HPDLF cell lines were obtained from the American Type Culture Collection (Manassas, VA, USA) and cultured according to their recommendations. The cells were grown in Dulbecco’s modified Eagle Medium supplemented with 10% fetal bovine serum and a 1% penicillin–streptomycin solution in an incubator (temperature of 37 °C, 5% CO_2_). The cells were seeded into 96-well plates (5000 cells per well). All assays were conducted using the same cell batches.

### 2.3. Evaluation of Cell Viability and Cytotoxicity of PSRE

The half-maximum inhibitory concentration (IC50) of PSRE was determined. The IC50 is the concentration at which 50% of the cells die. PSRE’s ability to inhibit biological and biochemical functions was analyzed. PSRE (dissolved in a 30% polyethylene glycol solution) was diluted in cell culture medium to concentrations of 25, 50, 100, 200, 240, 280, 320, 400, and 800 µg/mL and filtered through a 0,22 µm syringe filter. Cells were incubated with different concentrations of the extract for 24 h and then cell viability was assessed using the fluorescence microscopy method.

Cell nuclei were stained with the fluorescent dyes bisbenzinimide H33342 trichloride (Hoechst 33342) and propidium iodide (PJ). The cell membrane is permeable to Hoechst 33342, allowing it to stain the nuclei of viable and apoptotic cells, which then glow blue. The staining of the nuclei of viable cells is homogeneous and has smooth edges, while the stained nuclei of apoptotic cells are condensed and/or fragmented and glow an intense blue. The cell membrane is not permeable to PJ; therefore, only the nuclei of necrotic cells are stained with it and glow red. The cell cultures were incubated with 20 µg/mL Hoechst 33342 and 10 µg/mL PJ for 15 min. The samples were fixed with 4% paraformaldehyde for 10 min at room temperature to preserve the cell structures for microscopy.

Images of five random fields were captured using a Olympus IX71S1F-3 fluorescent microscope (Olympus Corporation, Tokyo, Japan) under 200× magnification. Viable, apoptotic, and necrotic cells were counted using Image J 1.53k software, and the average in five fields was recorded. Cell viability was expressed as a percentage of viable, apoptotic, and necrotic cells out of the total number of cells.

### 2.4. Analysis of Cellular Effects of PSRE and Chlorhexidine on HGF-1 Cells

The effects of PSRE and a 0.12% chlorhexidine digluconate solution (CHX) on the HGF-1 cell line were analyzed. The concentrations of PSRE used were lower than its IC50: 25, 50, 100, 200, and 240 µg/mL. The 0.12% chlorhexidine solution was prepared with the culture medium and filtered the same way as the PACN solution. The results were compared with two negative controls: growth medium (Dulbecco’s modified Eagle Medium supplemented with 10% fetal bovine serum and a 1% penicillin–streptomycin solution) and the 30% polyethylene glycol solution used to dissolve the PACN powder. Negative controls help eliminate the impact of external factors by providing a baseline for comparison.

The substances were incubated with HGF-1 cells for 24 h. The cells were monitored using the Prestoblue reagent and a spectrophotometer (Spark, Tecan, Basel, Switzerland). The Prestoblue solution was diluted 10-fold with the culture medium, and 100 µL of the diluted solution was added to the cells and incubated at 37 °C for 2 h. The Prestoblue method measures the metabolic activity of the cells. The resazurin contained in Prestoblue is converted into resorufin in the cells, the amount of which is measured spectrophotometrically at 570 nm with a 600 nm reference wavelength used for background correction. The amount of resorufin absorption is directly proportional to the metabolic activity of the cells, which, in this case, was used to reflect cell proliferation. The spectrophotometry results were recorded in relative units.

### 2.5. Evaluation of TGFβ-1 Levels in HGF-1 and HPDLF Cells

HGF-1 and HPDLF cells were treated with the controls or PSRE at concentrations of 25 and 50 μg/mL and proliferation was measured using an TGFβ-1 ELISA (enzyme-linked immunosorbent assay, Thermo Fisher Scientific, Waltham, MA, USA).

The ELISA was performed according to the manufacturer’s recommendations. The TGF-β1 ELISA uses specific antibodies to measure the concentration of this growth factor in biological samples. TGF-β1 regulates fibroblast function and therefore, these results can contribute to understanding the potential of using PSRE in periodontal disease treatment.

### 2.6. Statistical Analysis

The statistical analysis was performed using the IBM SPSS 20.0 (Armonk, NY, USA: IBM Corp.) statistic software package. For normally distributed data (tested with the Shapiro–Wilk test), one-way ANOVA was used to compare the means of the groups. The alpha level for significance was set to 0.05. The null hypothesis was that there is no difference between the groups regarding cell proliferation.

## 3. Results

### 3.1. Cell Viability and Cytotoxicity

The results showed that low concentrations (less than 100 μg/mL) of PSRE did not have an impact on cell viability while high concentrations (400 µg/mL or higher) strongly affected cell viability.

**Figure 1:**
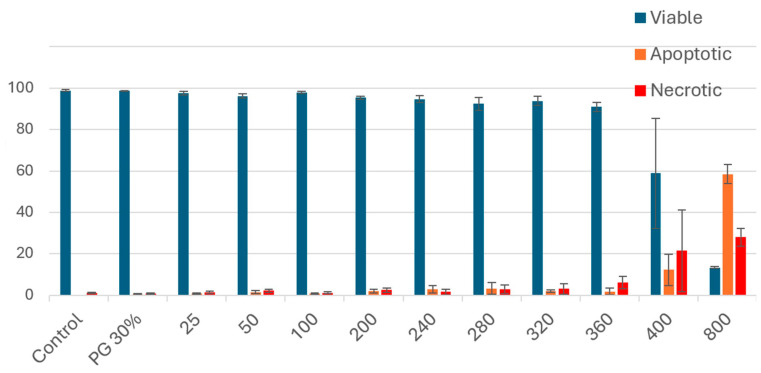
Determination of half-maximum inhibitory concentration of PSRE based on HGF-1 cell viability. PG 30%, 30% polyethylene glycol solution; concentrations of PSRE: 25–800 μg/mL; y-axis: % viable, apoptotic, and necrotic cells; *n* = 12.

The half-maximum inhibitory concentration was determined to be 583 µg/mL; therefore, the subsequent in vitro tests were performed with lower concentrations of the extract (Figure 1).

Previous in vitro studies used PACNs at concentrations of 10–50 μg/mL [32,33,34]. An in vitro study with fibroblasts confirmed that proanthocyanidins did not affect cell viability at concentrations lower than 100 μg/mL (after 24 h) [15]. Based on these findings, the PACN concentrations used in the subsequent experiments were 25 and 50 µg/mL.

### 3.2. Proliferation Potential of PSRE

**Figure 2:**
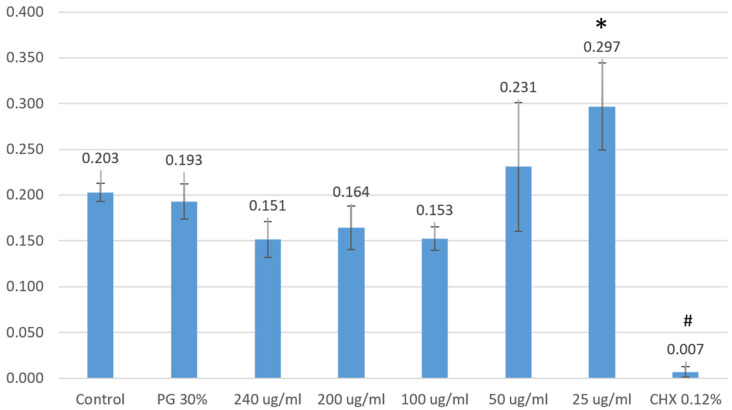
PSRE and chlorhexidine effects on HGF-1 cell counts (in relative units (mean ± SE)). PG 30%, 30% polyethylene glycol solution; CHX 0.12%, 0.12% chlorhexidine digluconate solution; PSRE concentrations: 25–240 μg/mL; *n* = 25. One-way ANOVA followed by LSD post hoc test: * control vs. 25 μg/mL (0.09 ± 0.03) *p* = 0.006; # control vs. CHX 0.12% (−0.20 ± 0.03) *p* < 0.001. Other groups showed no difference when compared with control.

The impact of PSRE and chlorhexidine on HGF-1 cell proliferation is shown in Figure 2.

The low concentration (25 µg/mL) of PSRE induced a higher level of HGF-1 cell proliferation in comparison with higher concentrations (100 µg/mL or higher).

In contrast, the 0.12% chlorhexidine solution inhibited cell proliferation completely.

### 3.3. TGFβ-1 Analysis in PSRE-Treated Cells

**Figure 3:**
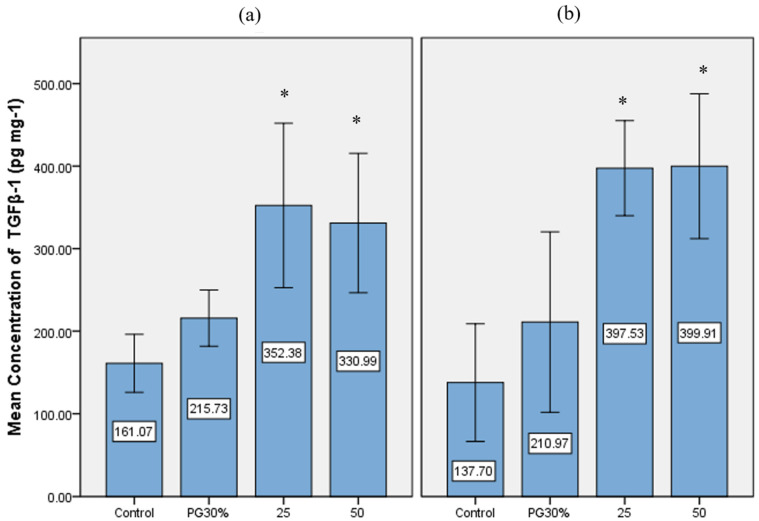
TGFβ-1 levels in PSRE-treated HGF-1 (**a**) and HPDLF (**b**) cells. Mean concentration of TGFβ-1 (pg/mL), *n* = 31. One-way ANOVA test followed by LSD post hoc test: * *p* < 0.001 compared with control. PG 30%, 30% polyethylene glycol solution; PSRE concentrations: 25–50 μg/mL.

The TGFβ-1 levels in PSRE-treated HGF-1 and HPDLF cells are shown in Figure 3.

There were significantly higher TGFβ-1 levels in the 25 and 50 μg/mL PSRE groups compared with the control group (mean ± SE) (352.38 ± 31.32 and 330.99 ± 26.53 versus 161.07 ± 11.04 pg/mL in HGF-1 cells; 397.53 ± 18.1 and 399.91 ± 27.61 versus 137.7 ± 16.54 pg/mL in HPDLF cells).

## 4. Discussion

This in vitro study evaluated the effects of PSRE on HGF-1 and HPDLF cells. The spectrophotometric evaluation and TGFβ-1 analysis showed that PSRE promoted fibroblast proliferation. Interestingly, the chlorhexidine digluconate solution inhibited cell proliferation completely.

Several PACN mechanisms of action are known, including modulation of intracellular pathways, such as the NF-κB (nuclear factor kappa-light-chain-enhancer of activated B cells), MAPK (mitogen-activated protein kinase), PI3K/Akt/mTOR (phosphoinositide 3-kinase/protein kinase B/mammalian target pf rapamycin), and NRF2/HO1 (nuclear factor erythroid 2-related factor 2/heme oxygenase-1) pathways [14,24,35,36,37,38,39,40]. A possible mechanism of action of PACNs is inhibition of the NF-kB signaling pathway through reducing the phosphorylation and degradation of inhibitor of kappa B [12]. Increased levels of inhibitor of kappa B bind to NF-kB and keep it in the cytoplasm, reducing its translocation to the nucleus [41]. This inhibition decreases the transcription of genes that promote cell apoptosis or inflammatory responses, creating a more favorable environment for cell proliferation [41,42]. PACNs can modulate the production of cytokines through the NF-kB pathway [43]. By reducing inflammatory cytokines (TNF-α, IL-1β), PACNs can decrease the inflammatory signals that might otherwise lead to cellular stress and apoptosis, creating a more supportive environment for healthy cell growth and proliferation. PACNs can reduce the levels of reactive oxygen species and prevent oxidative stress, leading to the suppression of NF-kB activation and preserving cellular functions that favor proliferation [44]. Other signaling pathways, such as the PI3K/Akt/mTOR and MAPK pathways, can interact with PACNs to cross-talk with NF-kB, further influencing its activity and the cellular responses.

Another possible mechanism of action of PACNs is activation of the TGF-β/Smad signaling pathway [45]. In fibroblasts, when TGF-β binds to its receptor complexes, TGF-βR1 (TGF-β type I receptor) and TGF-βR2 (TGF-β type II receptor), TGF-βR2 phosphorylates and activates TGF-βR1, initiating a signaling cascade: after rephosphorylation of Smad2/3, phosphorylated Smad2/3 forms complexes with Smad4 in the nucleus [45,46,47]. These complexes are the transcription factors that regulate type I collagen synthesis. After entering the nucleus, Smad4 induces collagen synthesis through transcriptional regulation [45,48]. PACNs may increase the level of TGFβ-1 [49], which can then stimulate fibroblast proliferation [50,51,52,53,54]. Therefore, the current study analyzed the TGFβ-1 levels in HGF-1 and HPDLF cells treated with PSRE as well. Interestingly, TGF-β can stimulate fibroblasts to produce bioactive factors (collagen, fibronectin, MMPs, tissue inhibitor of MMPs (TIMPs), and plasminogen activator inhibitor 1 (PAI-1)) that contribute to the deposition and remodeling of the wound extracellular matrix [55]. In summary, PACNs may increase cell proliferation and stimulate cells to synthesize collagen. Through these mechanisms, PACNs can have cell protective effects and regulate cellular growth, making them a potential therapeutic for chronic diseases.

Since periodontal regeneration is the restoration of the periodontium, which includes alveolar bone, periodontal ligaments, and root cementum [56], and fibroblasts play a significant role in these processes. Fibroblasts promote wound healing, tissue repair, collagen synthesis, and angiogenesis [57]. Fibroblast proliferation induces the formation of new tissues and restoration of damaged periodontal structures, providing a scaffold for new cells to migrate and proliferate on. Fibroblast proliferation is crucial for collagen synthesis, which is the main component of the extracellular matrix, which contributes to structural integrity, facilitates healing, and promotes the functional recovery of periodontal tissues, and is therefore essential for clinical periodontal regeneration. In agreement with the current study’s findings, other scientific studies have confirmed that PACNs, when acting on human gingival or periodontal ligament fibroblasts, exhibit cytoprotective effects, increase cell proliferation and migration, and increase collagen synthesis, which are responsible for the strong cell protective properties of PSRE [58,59,60]. Moreover, other studies have confirmed that PACNs can reduce bone resorption [20,61], improve bone metabolism [62], and increase bone regeneration [24,25,63,64,65], which are the key processes in periodontal tissue regeneration. Since periodontal ligament stem cells can differentiate into osteoblasts, and PACNs can promote osteogenesis of periodontal ligament stem cells, PACNs could have applications in alveolar bone regeneration [63,66,67,68]. There is scientific evidence that PACNs suppress osteoclast formation and differentiation while promoting osteoblast differentiation [69,70]. Our findings suggest that PACNs have the potential to enhance cell proliferation and viability.

Additionally, this study found that chlorhexidine digluconate (the gold standard treatment in periodontal therapy) completely inhibited cell proliferation. Other in vitro studies have confirmed that chlorhexidine digluconate should not be used during treatment as it may have negative long-term effects due to its established cytotoxic effect on oral cavity cells [71,72,73,74,75]. After periodontal treatment procedures, creating the proper environment for healing is crucial. PACNs could be used as an alternative to chlorhexidine in treating periodontal disease due to its similar antibacterial effect against oral microorganisms [76].

The current study had some limitations. A polyethylene glycol solution (PG30%, PG) was used as the solvent for PSRE. While PG solutions are water soluble and biocompatible, and have low immunogenicity, there is scientific data showing that there are dynamic biological interactions between PG and cells at the molecular level [77]. However, PG solutions are an effective solvent for the extraction and purification of PACNs [78]. Therefore, a PG solvent was used in this study but was tested on the cells as a PG-only control and no significant effects on the cells were found.

The proliferative effect of PACNs on different cell types must be investigated to determine whether this effect is specific to gingival and periodontal ligament fibroblast cells. However, an in vitro study using a single cell type limits the generalizability of the research results as multiple cell types interact in the oral cavity. Future research should test osteoblasts or 3D scaffolds to obtain deeper knowledge on PACNs’ cellular effects.

Potential in vivo or ex vivo studies could include analyses of the cell proliferation effects by measuring growth factor levels in gingival specimens or gingival crevicular fluid samples.

In conclusion, the proliferative effect of PSRE could play a significant role in the management of periodontal and peri-implant diseases [7].

## 5. Conclusions

The results of this study suggest that relatively low concentrations of PACNs may promote HGF-1 and HPDLF cell proliferation in contrast to chlorhexidine digluconate, which showed cytotoxic effects.

## Data Availability

The data supporting the present study can be obtained upon reasonable request.

## Abbreviations

PACN, (also PACNs)	proanthocyanidin (-s)
PSRE	*Pelargonium sidoides DC.* root extract
CHX	chlorhexidine digluconate solution
HGF-1	human gingival fibroblasts
TGFβ-1	transforming growth factor beta-1
HPDLF	human periodontal ligament fibroblasts
ELISA	enzyme-linked immunosorbent assay
SE	standard error
NF-κB	nuclear factor kappa-light-chain-enhancer of activated B cells
IC50	half-maximal inhibitory concentration
PI	propidium iodide
MAPK	mitogen-activated protein kinase
PI3K/Akt/mTOR	phosphatidylinositol 3-kinase/protein kinase B/mammalian target of rapamycin
NFR2/HO1	nuclear factor erythroid 2-related factor 2/heme oxygenase-1
TNF-α	tumor necrosis factor-alpha
IL-1β	interleukin 1 beta
TGF-βR1	TGF-β type I receptor
TGF-βR2	TGF-β type II receptor
Smad2/3/4	suppressor of mothers against decapentaplegic
PG30%	30% polyethylene glycol solution
